# “Using information to shape perception”: tobacco industry documents study of the evolution of Corporate Affairs in the Miller Brewing Company

**DOI:** 10.1186/s12992-022-00843-3

**Published:** 2022-05-21

**Authors:** Jim McCambridge, Jack Garry, Kypros Kypri, Gerard Hastings

**Affiliations:** 1 Department of Health Sciences, University of York, Seebohm Rowntree Building, Heslington, York, YO10 5DD UK; 2 University of Newcastle, Newcastle, Australia; 3 University of Stirling, Stirling, Scotland

**Keywords:** Alcohol industry, Tobacco industry, Alcohol policy, Corporate affairs, Alcohol education, Corporate social responsibility

## Abstract

**Background:**

The Miller Brewing Company (MBC) was wholly owned by Phillip Morris (PM), between 1970 and 2002. Tobacco industry document studies identify alliances between the alcohol and tobacco industries to counter U.S. policies in the 1980s and 1990s. This investigation sought to study in-depth inter-relationships between MBC and PM, with a particular focus on alcohol policy issues. We used the Truth Tobacco Industry Documents library to trace the evolution of corporate affairs and related alcohol policy orientated functions within and between MBC and PM.

**Results:**

MBC was structured and led by PM senior executives from soon after takeover in 1970. Corporate Affairs sought to influence public perceptions of alcohol to align them with business interests. Alcohol education was specifically designed to prevent the adoption of policies inimical to those interests (e.g., raising excise taxes). Strategic consideration of alcohol policy issues was integrated within company-wide thinking, which sought to apply lessons from tobacco to alcohol and vice versa. PM directly led key alcohol industry organisations nationally and globally, which have successfully delayed the adoption and implementation of known effective policy measures in the U.S. and worldwide.

**Conclusions:**

PM has been a key architect of alcohol industry political strategies. This study builds on earlier work on alcohol companies in the tobacco documents, and offers historical data on how tobacco companies have used commercial involvements in other sectors to influence wider public health policy. We are only beginning to appreciate how multi-sectoral companies internally develop political strategies across product categories. Global health and national governmental policy-making needs to be better protected from business interests that fundamentally conflict with public health goals.

## Background

The burden of harms caused by alcohol is large, avoidable, and expected to continue to grow globally [1]. National alcohol policies, where they exist, are under-developed and weakly implemented [1]. In contrast to tobacco, there is still widespread acceptance that alcohol industry actors have a legitimate role in the formation of public health policies [2]. This reflects an implicit view that alcohol companies differ in important ways from tobacco companies.

The Truth Tobacco Industry Documents library contains large volumes of internal tobacco company material that came into the public domain as a result of the Master Settlement Agreement of U.S. lawsuits against tobacco companies [3]. This archive has facilitated detailed understanding of the internal machinations of the tobacco industry, and served to strengthen tobacco control. There is no similar repository of internal alcohol company documents. In the context of the global burden of alcohol, the activities of major transnational producer companies are under-studied [4, 5].

The existing literature on alcohol companies based on the tobacco industry documents shows how useful is this data source. Jiang and Ling [6] illustrated how tobacco companies built coalitions designed to influence U.S. policies on taxation and other key issues by recruiting alcohol and other sectors in the 1980s. Jiang and Ling [7] also found that tobacco companies closely studied and sought to link tobacco and alcohol consumption. The Miller Brewing Company (MBC), owned by Phillip Morris (PM) from 1970 to 2002, was extensively involved in connecting the two [7]. Jernigan [8] identified MBC as key actors in the formation of the International Center for Alcohol Policies (ICAP), whose raison d’être was to counter the World Health Organisation (WHO) at a time when attention to alcohol policy issues was growing globally. Although the concept may be defined in different ways, subsequent study of this type of corporate social responsibility (CSR) organisation formed by alcohol companies draws attention to their activities in framing policy issues, the nature of the partnerships they form, and undeclared roles in seeking to influence policies [9–14]. Previous study of the relationship between MBC and PM has examined the PM CEO Issues Books 1996–2000 which set out company positions in advance of annual meetings [8, 15, 16]. Bond and colleagues [16] also identified evidence that PM Corporate Affairs (CA) and Worldwide Regulatory Affairs (WRA) provided direction to MBC during the 1990s, though there is no investigation of earlier precursors.

PM sold MBC to South African Breweries in 2002 to form SAB-Miller, and the PM U.S. parent company Altria retained a large shareholding until the 2016 combination with ABInBev. This merger, of the world’s two largest brewers, was then the third largest merger in corporate history [17]. Altria strongly supported the merger [18] and has retained a shareholding of approximately 10%, making ABInBev comfortably Altria’s largest external investment [19]. Altria also directly owns a wine producer, Ste. Michelle Wine Estates, and has a controlling interest in Cronos, a cannabis producer [19]. This reflects the breadth of Altria’s interests in a particular type of unhealthy commodity; drugs which are legal to consume [20].

This study aims to better understand how MBC operated within the PM group of companies, how it thought about and addressed long term strategic challenges, including in collaboration with other alcohol industry organisations. This study is explicitly descriptive in nature, being designed to add to, and strengthen, the earlier work on alcohol companies based on the tobacco industry documents. Undertaking this study develops potential for advancing understanding of the nature of inter-relationships between tobacco and alcohol companies, and how multi-sectoral companies internally develop political strategies across product categories. More sophisticated understanding, drawing on appropriate theoretical frameworks, may in turn inform the further development of public health policy making [4].

## Results

### Leadership and organisation

PM Incorporated bought MBC outright in 1970 [21, 22]. Senior personnel movements provide indications of how leadership was exercised. John A. Murphy originally joined PM International (PMI) in 1962, where he was a Vice President (VP) [23, 24], before becoming Chief Executive Officer of MBC in 1971. He subsequently became Executive VP for PM Inc. in 1976, where he was responsible for non-tobacco operations, and later served as President and Chief Operating Officer for the entire PM Inc. group of companies from 1984 [22, 25–27]. John D. Bowlin worked his way up in marketing in PM food companies between 1974 and 1993 and had two spells at MBC, taking over for 16 months as President and Chief Operating Officer in 1993–94 and later from 1999 as President and Chief Executive Officer [28], in both instances when PM company leadership was dissatisfied with MBC business growth. In a strategic overview in 2000 [29] he specifically identified the benefits of “*recruiting and seeding Miller with first rate executives from our other Philip Morris companies*.” Senior personnel movements were not all in one direction, however, and included the key CA function. For example, Guy Smith was VP CA for PM Companies Inc. from 1989, after starting his PM career in MBC [23]. Decision-making within MBC was, as will be seen below, nonetheless shaped profoundly and directly by PM executives.

MBC organised CA in a tripartite structure from 1981 onwards, with the VP CA leading Directors of Government Affairs, Corporate Communications and Communications Services departments [30]. Across these departments, in the context of a statement of company concerns on regulation and public opinion, CA was stated explicitly in the 1981 as engaged in:

“*presenting the company's position as a supporter of education and research to combat the problem of alcoholism rather than imposition of additional restrictions on the use of alcoholic beverages*” [30].

This conception of the major strategic policy challenge facing the industry, and the nature of the response needed, closely mirrored that developed by the public relations company Hill and Knowlton for the tobacco industry over many years [31, 32]. This included the writing of the original “A Frank Statement to Cigarette Smokers” and setting up the Tobacco Industry Research Committee and they also developed the same approach for the U.S. spirits industry [33].

The MBC structure sat within the functional direction of the overarching PM Inc. group of companies, which was organised to include centralised marketing, legal, financial, and CA functions [34]. The PM leadership became concerned about what they regarded as: “*the increasing influence of the anti-drinking movement*” by the April 1984 PM annual meeting [35], and MBC developed the company’s own programme in opposition to it, alongside beer and alcohol industry-wide ones [36].

### The nature of MBC corporate affairs strategies in the 1980s

CA was of vital importance in developing MBC strategies bearing upon policy. Alan Easton was the MBC VP CA from 1981 or earlier [30]. Easton elaborated on the nature, activities and goals of CA as set out in Table 1 [37]. The opposition to evidence-based population-wide control measures on taxation and marketing was typical of other parts of the alcohol industry in the U.S. at the time [40], and this continues to be the case for the alcohol industry globally, which advocates education, voluntary and targeted measures instead [41]. There were strong similarities in the ways in which health and science posed issues for alcohol and tobacco, and both necessitated coalitions [38, 39]. The internal company documents reveal that at least as early as 1984, MBC understood the long term strategic threat to its business posed by scientific evidence on alcohol, and had decided at a high level how it should respond.

**Table 1:** Alan Easton, Miller Brewing Company VP Corporate Affairs Perspectives in 1984

*From speech to Miller Management Club, October 1984* [37] “Basically, Corporate Affairs means getting and using information to shape perception. That’s what we’re all about. Information shaping perception. Although we use different nomenclature, part of our operation is what most people call public relations. Another of our functions is that of the lobbyist. And both PR and lobbying are terms with serious image problems.” “Corporate Affairs exists... ... to protect Miller’s freedom to conduct business, and conduct that business at a profit; ... to get many publics favorably disposed toward Miller and our products — and keep them that way; ... and to help sell beer, by helping establish brand awareness, by reinforcing Miller advertising and promotions, by vastly expanding the audiences exposed to them, and by making advertising and promotion dollars go farther and do more” *From transcript of speech to the Phillip Morris Inc Board, December 1984* [38] “The battle lines (sic) are clear. And our battle plan has been carefully drawn…Our battle will be waged largely through a broad-based excise tax coalition, with the brewers at the apex…Wine will be a strong and effective ally. Distilled spirits, perhaps less so, but still important. Beyond the direct industry participants, certain supplier industries will be mobilized…(emphasis in the original) [on advertising] this effort will not be short term. This battle will continue to be a continuing one and may ultimately find its way into the courts. The constitutional questions under the 1st and 4th Amendments are considerable and we’re already mapping our legal position. Like foresters who plant seedlings now for future harvesting, we’re putting our seeds in the ground right now. At Miller we are taking the initiative. We are not waiting, until these issues are upon us.” (emphasis in the original) *From formal report to the Phillip Morris Inc Board, December 1984* [39] “the establishment of industry coalitions required in connection with voluntary industry advertising guidelines, federal excise tax proposals, and consumer activist demands for a ban on broadcast advertising and other restrictions on marketing… Financial and other support of key national anti-alcohol abuse programs and organizations which are consistent with Miller’s pro-education/anticontrol position will be expanded.”	

### The position of MBC within PM in the late 1980s and early 1990s

At the PM company-wide international CA conference of 1986, the year the U.S. National Minimum Drinking Age Act was implemented [42], Hugh Cullman, Vice Chairman, PMC Inc. described the alcohol threat as:

“*a new and active temperance movement whose first step has been to raise the drinking age…In beer, the demands for controlling sales and advertising are scapegoats for inadequate social, medical, and educational efforts to deal with alcohol abuse… the most important thing to keep in perspective is that the products we produce are basic sources of human pleasure and satisfaction*” [43]

Despite existing appreciation of the nature of the threat posed by alcohol policy measures, the issues appeared to carry limited prominence in the thinking of the PM leadership about overall strategic issues during that decade, according to coverage within key documents. This was likely due to the growing salience of the emerging threats to the tobacco side of the business. The bulk of attention to alcohol in the strategic planning documents is to market expansion plans, very largely within the U.S., in line with the history of MBC. For example, the PM chairman briefing on preparations for the 5 year plan for 1989–1993 following the passing of U.S. legislation on warning labels in 1988 [44], states simply;

“*legislative Environment: Plans relative to warning label requirements and potential excise tax increases*” [45]

PM lobbying of members of congress on key committees was well organised and involved a division of labour according to pre-existing relationships across the group of companies as a whole on cross-cutting issues [46]. The PM Board’s oversight of the political issues faced by MBC was thus integrated within company-wide thinking and included giving direction. A 1991 PM strategic review linking weak business performance and potential regulatory threats identified priorities for 1992 such that;

“*Miller should take the lead in industry efforts to prevent an advertising ban or other marketing restrictions. We are not in a good position to take a ban*” [47]

An example of successes in PM lobbying on inter-linked alcohol and tobacco issues was reported by Craig L. Fuller, Senior VP PM CA early in 1992;

“*We were able to delay a February markup in the Energy and Commerce Committee on the re-authorization of the Alcohol, Drug Abuse and Mental Health Administration (ADAMHA) in order to negotiate acceptable tobacco and alcohol provisions. The Subcommittee's report included provisions for a national minimum sales age of 18 for tobacco products and a “gateway” drug study involving tobacco and alcohol. The industry-sponsored compromise dropped the gateway drug study and adopted language, which we drafted, narrowing the HHS Secretary's enforcement authority. That compromise passed the full committee*” [48]

As the 1990s progressed, the looming threat of tobacco litigation appeared to dominate strategic thinking about the future of the business. In April 1994 PM was considering selling off Kraft General Foods, in the context of the challenging tobacco issues it then faced [49]. While PM recognised the possibility of a future sale of MBC, it decided to pursue global expansion of the alcohol business at that time [49].

The position of MBC as an integral component of the PM company is exemplified in how PM mobilised MBC employees politically. From 1982 or earlier the PM leadership encouraged MBC employees to be involved in fighting federal cigarette excise tax increases by contacting their local representatives [50]. MBC Chairman John MacDonough wrote directly to MBC employees on tobacco in 1994 [51], and such efforts continued throughout that decade [52, 53]. The consolidation of the Political Action Committees (PAC) of MBC and PM in 1984 [54] further reinforced the recruitment of MBC employees to lobby on tobacco-related issues [55]. In the 1995 PAC fundraising tour of all operating companies by Geoffrey C. Bible, the PM President and CEO noted that:

“*the future of our businesses -- whether food, beer or tobacco -- depends to a great extent on a political environment in which we can pursue those businesses profitably*” [56]

### PM-led activity in formative years for the alcohol industry globally in the mid-1990s

From April 1993 Tim Scully was the Director of Washington Relations for MBC [57], though he reported directly to PM VP Government Affairs, rather than being located within the MBC structure [58, 59]. At this time the PM CA structure comprised Corporate Public Affairs, Government Affairs, Corporate Communications, and CA policy and administration. Scully worked within the Washington Relations Office (WRO), which also contained a Kraft VP [60]. Scully’s responsibilities in 1995 are mapped out in detail in Table 2 [61]. He had a trade union lobbyist background with the Teamsters [57, 62]. When newly introduced “Issues Management” staff were interviewing key figures in 1995, he was seen to have an interesting:

“*perspective on the ‘do-gooder’ groups. Doesn't want to tear them apart, Instead, looking for ways to develop ‘quiet’ relationships*” [62].

He was also noted to be encouraging of direct contact on issues management with Anheuser Busch (the leading domestic competitor) whom he regarded highly [62]. Scully was promoted to VP Federal Government Affairs for MBC in 1996, and stayed within the PM structure until after 2000 [57]. By 2000 it was stated that Scully “*manages all federal and international governmental affairs*” and was an ICAP Board member [57]. He worked for MBC after the sale by PM, and at his retirement in 2018 was noted to have been on the Beer Institute’s Management Committee for nearly 20 years [63]. Mike Jones, Associate General Counsel and Assistant Secretary of MBC was also an ICAP Board member in 2000 [28]. PM, via Scully and others, were thus directly involved in the formation and early years of ICAP (see below), the key alcohol industry body globally.

**Table 2:** Executive Department, Congressional Committee, Issue and Organizational Assignments For Tim Scully [61]

**General Issues** Responsible for all issues in the Executive Branch and the Congress that concern the Miller Brewing Company. **Congressional Committees and Executive Branch** • Commerce Committee, U.S. House of Representatives • Transportation and Infrastructure Committee, U.S. House of Representatives • Ways and Means Committee, U.S. House of Representatives • Economic Opportunities Committee, U.S. House of Representatives • Commerce, Science, and Transportation Committee, U.S. Senate • Environment and Public Works Committee, U.S. Senate • Finance Committee, U.S. Senate • Labor Committee, U.S. Senate • U.S. Department of Treasury - Bureau of Alcohol, Tobacco, and Firearms • U.S. Department of Health and Human Services - Substance Abuse and Mental Health Services Administration - Center for Substance Abuse Prevention - Center for Substance Abuse Treatment • Federal Trade Commission **Specific Issues** (A = active, D = dormant) Federal Alcohol Excise Taxes (A) Tax Deductibility of Advertising Costs for Alcohol Beverages (A) Other Tax Proposals Impacting on the Beverage Industry (D) -FICA Taxes on Tips -Business Meal Deductions Malt Beverage Interbrand Competition – territorial franchising (D) Alcohol Liability Laws (D) Ad Bans Mandatory Advertising Warning Messages (A) National Minimum Blood Alcohol Content (A) National Minimum Drinking Age (A) Alcohol as Drugs (A) Labeling Requirements (A) Advertising and Use of Athletes (A) Container Fees and Mandatory Container Recycling Requirements (A) Clean Water Act Reauthorization (A) Superfund Reauthorization (A) EPA Classification of Hops (A) Regulatory Moratorium (A) Regulatory Reform (A) Product Liability (A) General Labor Law Issues (A) - Striker Replacement Legislation - Teamwork - Revision of the National Labor Relations Act - OSHA Reform **Outside Groups** Beer Institute National Beer Wholesalers Association Distilled Spirits Industry Council Wine Institute Association of National Advertisers AFL-CIO and its Affiliated Unions (BCT, Teamsters, Machinists, UAW) National Restaurant Association	

The Beer Institute was and remains the main U.S. domestic beer trade association, and e-mail correspondence reveals how it was run by the major companies. For example, the modus operandi in 1995 was such that;

“*any one member of the management committee (MBC, Anheuser Busch, and Coors) can veto the BI's involvement*” [64]

This was important as not all of the Beer Institute’s activities were confined to beer; PM also enlisted it to support on tobacco [65], including to “*help us quietly or even in the open*” [66].

Scully was active also with other domestic alcohol industry organisations, for example meeting with the Brewers Association of America which represented small brewers; “*to bring the Association’s views in closer fit with MBC’s and the Beer Institute*” [67]. Scully also worked closely with David Nicoli, a tobacco specialist within PM, and indeed also himself worked on tobacco [62], while Nicoli was reciprocally involved in alcohol [66]. Interactions included communications with regulators on emerging and sensitive business issues, with Scully reporting for example;

“*We also began discussions with ATF [Bureau of Alcohol, Tobacco & Firearms] over the possible introduction of other flavored malt beverages like alcoholic lemonade and iced teas. This is a very controversial area with the ATF because of the age marketing implications*” [67].

PM’s steering of MBC involved discussions among a range of PM Management Corporation CA staff that did not involve MBC leadership or MBC CA, including in mobilising the Beer Institute and other beer industry actors [68].

MBC’s involvement in establishing ICAP in 1995 has already been identified to have embraced a global perspective on issues management that serves the internationalization of the business according to the CEO Issues book [8]. This was also reported in the Phillip Morris Globe newsletter, December 1995 as follows;

“*Miller’s long term business success and its viability as a leading international brewer depends on how well it competes in the constantly changing and rapidly expanding global economy. Since international issues will help shape Miller’s strategies and tactics in the worldwide beer marketplace, issues management plays a key role in Miller’s plans. Miller’s involvement in ICAP, as witnessed by the success of its September meeting, is a significant first step for the company in improving its capability to manage alcohol issues on a worldwide basis*” [69].

In July 1996, Patti McKeithan the MBC VP CA set out to an industry audience the company’s strategic conception of alcohol education, specifically how it was designed to counter alcohol policies (see Table 3) [70]. Contrary to claims that industry actors make publicly about the independence of organisations such as ICAP, this presentation also elaborates on the actual rationale for ICAP [70].

**Table 3:** Extracts from; “Alcohol Education: An Essential Factor in Preserving the Alcohol Beverage Industry” Delivered by Patti McKeithan, MBC VP CA to an industry group, July 1996 [70]

I believe the number one priority for the alcohol beverage industry… [all … in original unless stated] over the next five years…must be protecting and promoting the social acceptability of our product. Alcohol education will play a critical role in accomplishing this task. I am using the term “alcohol education” in its widest sense…and we must think in terms of at least three audiences: First, we must continue to educate consumers to drink our products responsibly. Miller believes this kind of education is vital…so we have developed… and put in place…a wide variety of programs covering: • Training of servers, • Training of sales people in prevention of underage sales, • Designated driver programs, and many others. You will find brochures…in the back of the room giving much more information on these programs…and how you can take advantage of them in your own efforts. Second, we must continue to educate the public…that there is a vast difference…between consumption…and abuse…of our products…and between alcohol…and illegal drugs. We are dedicated to finding and adopting creative and effective solutions to alcohol abuse. Introducing alcohol awareness curricula in schools, broadcasting public service announcements for drivers, and funding research are only a few examples of the many ways we accomplish these goals. We are fighting alcohol abuse…because no reputable business benefits when its products are misused…and because the families affected by alcohol abuse are our friends, our neighbors…and our families too. And third, we must continue to educate policy makers …that we…and the 100 million Americans who drink alcohol beverages…don’t need higher taxes…and more restrictive regulations…and that we must avoid simplistic, shotgun approaches to complex problems. …[later in the speech] With the globalization of our industry…and the globalization of the anti-alcohol and public health community…Miller Brewing Company believes the industry must speak with one voice on policy, globally. For that reason, in December of 1994, Miller Brewing Company and nine other major world producers of beverage alcohol founded the International Center for Alcohol Policies (ICAP), based in Washington, D.C. This organization’s goal and aim is to help reduce alcohol abuse worldwide…to promote understanding of the role of alcohol in society…and to encourage dialogue…and pursue partnerships between the beverage alcohol industry and the public health community. In the short time since its inception, ICAP has exceeded its most optimistic goals. …[text omitted] That’s why we’re going to become more involved with the government organizations that set policy and fund programs related to alcohol. Armed with the facts…and the Federal Government’s own policy…we are going to put an end to the untrue…unfounded…and unfair attacks on our products. Make no mistake…these flawed policies have been intentionally foisted on us…and on these agencies…by the anti-alcohol forces. It’s time to correct this injustice…and we will use the government’s own policy and science to do it. Science is on our side. Truth is on our side…and if along the way…we get some coverage in the media…that will only help to remind consumers that alcohol beverages can be part of a healthy diet.	

### PM worldwide regulatory affairs and corporate affairs perspectives relating to alcohol in the late 1990s

WRA was formed in 1994 to address regulatory issues nationally and internationally in relation to tobacco, food, and beer within PM [71]. In 1995 it hoped to develop;

“*Better tracking of regulatory issues, especially the need to anticipate emerging issues before they become a problem. Good research and analytical capability on social science issues and on advertising and marketing stuff, including a good mastery of the literature*” [71, 72].

WRA was a separate unit to CA (within which Issues Management was originally located), and both were located within the legal department [73].

The PM approach to MBC around this time included identifying lessons that were applicable to other PM companies as the problems caused by tobacco litigation mounted [74]. For example, Steve Parrish, Senior VP CA, reported to the PM Board in April 1995:

“*both Kraft and Miller have developed some outstanding issues management programs and the question became how to extend these learnings, systems and programs so that they can be applied in all geographies where such application would be effective. WRA is taking a liaison role, bringing the various players across companies together and creating the networks and communication vehicles that will facilitate the sharing of valuable information on issues management* [75]

There were strong continuities in the basic lobbying stance of MBC itself over time. For example, in 1996 MacDonough wrote to a congressman that;

“*we share your desire to reduce underage consumption of beverage alcohol products. We also believe, however, that this goal can be achieved and, in fact, is being achieved by the industry itself in the absence of additional legislation*” [76].

Rhetorically, distancing MBC from PM was accomplished in the following terms;

“*there are differences between the two industries. As you are aware, the federal government itself has stated that moderate use of alcohol is part of a healthy life style. The beverage alcohol industry is subject to an extensive and specific regulatory framework and is subject to review by various government agencies*” [76].

However, PM actually directly guided MBC political activities in numerous ways. For example, campaign contributions were formulated on a state-by-state basis targeting both Democrats and Republicans by PM, as seen in communications from Scully [77].

Senior company leadership thinking in 1997 about the strategic policy issues to be managed in the U.S. continued to prominently feature the demonstration of concern about the issue at hand, and working closely with industry allies [78]. Around the end of 1998, it was recognised by PM that the value of; “*Miller programs on drunk driving, youth drinking are clearly identified and respected*” and these were seen as important to PM as a political player. This helped PM efforts to represent themselves as the “*good guy*” among the tobacco companies following the various legal and related proceedings that had damaged tobacco industry public relations [79]. By this time, PM direction of MBC was so advanced that PM Management Corporation saw itself as “*the CA Department*” for Miller [79], with the emphasis clear that this was one company with different operating units [80]. By 1999 there were lessons to be learned from the tobacco experience for PM that were applicable to alcohol (and food), for whom the question “*can the trial lawyers be far behind*” [80] was posed (see Table 4). This content was also delivered directly to MBC audiences [81, 82].

**Table 4:** Lessons from tobacco for alcohol

“First, do not simply reject or ignore criticisms… Second, pay close attention to public concerns, and most importantly, address those concerns… The third lesson we’ve learned is that we must understand the individuals and groups who are driving public opinion. When you look for common ground with your critics, you divide them into two different categories: the reasonable and the unreasonable. Then you will know with whom you can work, and with whom you cannot. Finally, we have learned that it’s a mistake to climb into a bunker when under attack. You must reach out, engage your critics, and speak often and honestly. If people stop seeing your face, your opposition can demonize you” [80]. *4 Strategic directions (our italics)* “*Constructive Engagement* on our issues - listening, seeking reasonable solutions, fighting only the right battles. *Societal Alignment* - working systematically over the long run to make sure that we are and are seen as a responsible manufacturer and marketer of all our products. *Proactive Dialogue* - an overall stance of reaching out, listening, talking, and engaging with others. *Image Enhancement* - long range efforts to increase our visibility and improve perceptions of who we are. All of these are long-term initiatives” [80]. *The emphasis in the third strategic direction in relation to strategic alliances;* “Proactive Dialogue - involves interacting more aggressively with the outside world. We define that as forging new alliances. Integrating with important associations and groups. Devoting more time to external affairs. And seeking input from others about our issues and programs. While Corporate Affairs will lead this effort, ultimately everyone in management has a responsibility to connect with their constituencies in tangible ways” [81].	

PM CA noted that MBC leadership had identified alcohol policy strategy as a high priority for the year 2000 and had created a task force [83]. Looking ahead to 2001, PM CA identified for MBC the four key issues facing them in the following terms:

“*Social cost arguments; Industry in-fighting; Resource constraints v. opponents’ resources; Uneducated media/ emotionalism of issues*” [83]

By late 2001 there were reports that PM was preparing to sell MBC for reasons of long term under-performance, and there was a high level of awareness within the company of the profound nature of the public relations challenges that were being faced; these included awareness of conflicts between stances on under-age tobacco and alcohol use [84]. MBC was sold off to South African Breweries in 2002 to form SAB-Miller.

## Discussion

Ownership of MBC entailed full control by PM, and the extent of commitment to, and level of direction of, CA and interventions in alcohol policy issues increased over time. This perhaps resulted from increased salience, and as lessons were being drawn from the evolving tobacco experience. The core features of the approach to alcohol policy issues were very similar to the approaches developed for tobacco, and exhibited strong continuity over time, and indeed persist to the present day [10, 13, 41, 85–89]. PM has been central to the leadership of alcohol industry alliances, directing US national trade associations and ICAP, and also has used involvement in alcohol in furthering its core political interests in tobacco. The key public policy-related management function was located within corporate affairs, in turn located within the legal department of the parent company. Although the organisational arrangements evolved significantly over time, the strategies for opposing evidence informed policies were highly consistent. As the strategic issues posed by both product categories overlapped [90, 91], it was logical to manage them centrally within the company, and future litigation was anticipated for alcohol, just as it was for tobacco. These findings add substantially to earlier investigations, which identified data that was suggestive of the deeper relationships uncovered here [6–8, 15, 16].

Strengths of this study include in-depth collection of internal company documents over an extended period and the construction of a chronologically ordered account of the development of key ideas and inter-relationships between MBC and PM. The nature of the tobacco company documents library means that the data on which this study is based allow scope for inferences on strategic thinking, and note this report relies extensively on direct quotations. Among the limitations are that data saturation cannot be claimed to have been reached, as many issues over many years are covered, and also that the data examined are dated. This makes important careful consideration of the historical context in which the relationships described are located, as well as complicating analytic attention that is directed towards the implications for the present day. Further documentary study can be expected to yield additional insights, though it seems unlikely that these would undermine the validity of the present findings. The present study serves to elucidate the feasibility and potential significance of more in-depth analyses of data on MBC/PM in the tobacco document archive, for example on framing. This is an important preliminary study of one company in the history of the political organisation of the alcohol companies during a key period in the globalization of the industry. We suggest that even though this descriptive study may be modest in nature, the importance of the data suggest there are clear contemporary policy implications.

MBC was unusual as a major alcohol company wholly owned by a tobacco company, but was certainly not unique [92]. It is thus worth considering carefully both the external validity of these data in relation to other alcohol companies, and what they tell us about the relationships between the two sectors. Major alcohol companies globally have now worked closely together in ICAP and its successor organisation (the International Alliance on Responsible Drinking) for a quarter of a century, with strategic policy positions very similar to MBC/PM [8, 10]. These have changed little over several decades [40, 41]. This study reveals the backstage operations of this one company, whilst CA and CSR are practised in public in highly similar ways in, and indeed with, other alcohol companies.

The fiduciary imperative requires that major US (and UK) companies put shareholder interests first; interests that appear defined by this company’s leadership in narrow profit terms. Within the company these were understood to be best served by the preservation of “freedom to conduct business” by fighting off unfavoured policy proposals (see Table 1). The literature on how the tobacco companies have sought to shape processes of globalisation to advance their interests is most developed [93, 94]. The alcohol policy implications of the evolution of multi-level governance regimes and alcohol company responses have begun to be studied [95, 96]. Opposing effective policy measures entails disregard for the disease, violence, injury, and death caused by use of alcohol products, other than as presenting issues to be managed along the way, and viewing the scientific study of these issues as a threat to business interests. The ethical and scientific issues raised by this approach are well understood in relation to tobacco [97], and evidence on alcohol research is emerging much more recently [98–106]. These studies show how highly policy relevant alcohol science has been undermined in a range of ways which are similar to those used for tobacco. These observations likely apply also to the food operations of the particular company studied here in relation to obesity, though dedicated study is needed. After Kraft was sold by PM it merged in 2015 with Heinz, for whom 3G Capital has been a major shareholder. 3G Capital is also the major shareholder in ABInBev, and Altria the second largest. The question remains how far one may generalise inferences from the data on MBC, PM/Altria and tobacco examined here.

It appears that alcohol “education” was promoted not because there was any reliable evidence showing its effectiveness, but as a means of avoiding policy measures which presented fewer constraints on business [30]. This focus also presented a means with which to engage policy makers as shown in the detailed content of Tables 1 and 3 (together these provide an elaboration of the “pro-education/anticontrol position” rationale and approach)*.* The research consensus has long been that alcohol education is too weak an intervention to reduce the societal burden alone, and that regulation of the market is needed [107]. The fiduciary imperative may be interpreted differently in US and UK companies than in German or Japanese ones because they are constituted in distinct ways, and there are different expectations of their conduct [108], though competitive pressures may encourage a narrow focus on shareholder returns.

As with cigarettes, there is no safe dose of alcohol and consumption involves risk, which rises with level of consumption [109]. The use of any alcohol products is thus inherently risky. Individuals can choose to manage their use in different ways, and use generates an aggregate burden of mortality and morbidity at the population level. From a societal perspective, the tobacco and alcohol industries present a threat to public health in proportion to the extent of use of their products. From a commercial perspective there is also a shared threat, but in the opposite direction, in the form of public health regulation to contain harms intrinsic to both products. At this key strategic level, it did not matter whether MBC/PM produced cigarettes or beer, competition was set aside and alliances formed with other companies. The evidence in this study shows that these collaborative efforts sought to ensure that policy and regulation would not run contrary to business interests. Business interests are of course entirely legitimate to pursue, and the concerns raised by this study are to do with the content and manner of the claims being made by major alcohol and tobacco companies, and the consequences of their actions for global health and society. Smaller companies may be more responsive to societal concerns than bigger ones [110], and their interests may be submerged in the domination of trade associations by large companies, as seen here. The MBC/PM strategy in respect of education, and the ways in which it has been discussed within the company and presented to another industry group, appear very similar to the approach developed in the U.S. distilled spirits part of the alcohol industry [33]. There it was explicitly discussed in public relations terms, and although that term was used within this company, it has been seen here to be discussed within the organisational context of CA.

The importance of preventing collaborations between companies that produce health damaging products, and preventing their influence on public health policy, is well understood in relation to tobacco [31, 111]. Article 5.3 of the Framework Convention on Tobacco Control states further that signatories shall protect public health policy making relating to tobacco control from the commercial and other vested interests of the tobacco industry [112]. This study draws attention to the lack of similar consideration of this key issue for alcohol [113]. It is anomalous that tobacco control is specifically protected from Altria’s influence, whilst alcohol and wider public health policy making is not [114]. The same point could be made about science policy [113]. The implications of regarding alcohol companies and organisations created by them as partners in alcohol policy making [115] are readily apparent in the global burden of alcohol disease and injury [109]. Alcohol industry involvement in policy making responding to alcohol harm in public health and society has not been denormalized in the ways that tobacco industry involvement has been. There has been no progress in reducing per capita consumption globally after a decade of efforts, and the situation is expected to get worse unless stronger policies are implemented [1]. ICAP has been successful in countering WHO in that it has delayed the implementation of alcohol policies across the world [8]. The need to accelerate action to reduce alcohol harms has now been recognised by WHO, and alcohol industry interference in policy making has been identified as a key obstacle to be overcome, along with the harmful effects of alcohol marketing [116].

Study findings also make clear the limitations inherent in identifying corporate sectors according to the nature of their products rather than focusing on company ownership and leadership, and how interests are defined and advanced. This is especially so as the products of any one company often cross several categories, with many major alcohol producers for example, also involved in non-alcoholic beverages [117], and vice versa, e.g. [118]. Multinational corporations are increasingly global in outlook and expand horizontally as well as vertically.

This study contributes to the emerging literature on the commercial determinants of health [119–121] in other ways. There are business models available which describe how profit may be pursued alongside social objectives [122, 123], though whether this will be pursued by alcohol companies is moot given that their growth and profitability rely on increasing consumption, with deleterious consequences for global health [124]. The present findings are in line with existing evidence that alcohol industry CSR is unlikely to contribute to reducing alcohol harms, because as other studies have found, alcohol industry CSR is not actually designed to do so [13]. Indeed companies that produce intrinsically harmful products more broadly may make public health and environmental problems more intractable by impeding policy development [125], as existing evidence on tobacco and alcohol companies shows [41, 111]. The key inference to be drawn from this study is that the claims of major companies should be assessed not on the basis of what they say they are doing in CSR and CA, but by rigorous study of their conduct, particularly including whether and how they interfere with policy making. The key implication of this study is that, until such companies are fundamentally reformed such that the protection of public health and social welfare defines the scope for maximizing shareholder returns, they should not be allowed anywhere near the policy making process. It remains unclear how far and in which ways governance of such companies in the public interest may be attained.

## Conclusions

Little is known about the formation and nature of inter-relationships between tobacco and alcohol companies. Similarly, how tobacco companies use commercial involvements in other sectors to influence wider public health policy is largely unstudied and we are only beginning to appreciate how multi-sectoral companies internally develop political strategies across product categories. This study makes an important contribution in this context. PM directly led long term MBC and wider alcohol industry political strategies in the U.S and globally. PM also utilised alcohol industry organisations to bolster its defences against tobacco control policy. Global health and national governmental policy-making needs to be better protected from business interests that fundamentally conflict with public health goals.

## Methods

Methods for collecting and analysing data in the Truth Tobacco Industry Documents library have developed over time [126]. We adopted standard approaches, starting with a first wave of searches to scope the availability of information relevant to the research aims, then undertaking subsequent waves, snowballing from earlier sources to follow up on the most promising lines of enquiry. We compiled lists of names of key individuals and roles in organisational charts (see Table 5 for a summary of searches undertaken). Using the basic search facility, JG performed searches without restrictions on time, document type, or any other parameters. After screening, documents returned were read, downloaded and those retained printed, with notes on each document. All documents were read by JM, who reduced the dataset for more in-depth study, and undertook further searches. The analysis draws heavily on internal company accounts of the activities and relationships being studied, with themes developed by first and second authors. Note the approach taken was concerned with the manifest content of the documents examined and we have eschewed any discourse analytic or other in-depth theoretically governed method of analysis. This material was checked and triangulated with external data sources at various junctures to provide context, and to appraise the validity of the content.

**Table 5:** Summary of searches

Searches run in the Truth Tobacco Documents Library used various search terms in the basic search facility, generally with no restrictions on time, document type or other parameters. First, there was a preliminary scoping stage in which we established appropriate search strategies, and identified material of substantive interest. After preliminary searches were completed we used organisational charts to identify key roles of interest, and the names of the individuals occupying these roles at different points in time. Where ‘Organizational chart’ filter search was applied, it was as follows: documentdate:[19700101 TO 20091231] AND “miller brewing company” The main block of searches were run as they are presented below, with the boolean operator ‘AND’ used to link to Miller OR Miller Brewing Company where appropriate. This stage involved screening 1999 records and retaining 269 full text papers, all of which were downloaded and examined by JG and JM. The final stage involved snowballing from this dataset, following up on the most promising lines of investigation. As the screening of documents was done purposively in this final stage, we do not report the additional numbers screened or retained for further analyses.			
**Individual named actor searches**			
Search term	Hits	Screened	Retained
(“John A Murphy” OR “John Murphy”) AND “Miller brewing company”	1054	200	33
(“William K Howell” OR “William Howell” OR “Bill K Howell” OR “Bill Howell”) AND Miller	606	200	9
“Warren H. Dunn” OR “Warren Dunn”	352	200	11
“Lauren S Williams” OR “Lauren Williams”	335	200	5
“Leonard J Goldstein” OR “Leonard Goldstein”	359	200	6
“Alan G Easton” OR “Alan Easton”	181	181	52
“Clifford R Williams” OR “Clifford Williams”	160	160	3
“John J McGrath” OR “John McGrath”	239	239	3
“Obrie Smith”	63	63	4
(“jack n macdonough” OR “john n macdonough” OR “jack macdonough” OR “john macdonough” OR “jack n mcdonough” OR “john n mcdonough” OR “jack mcdonough” OR “john mcdonough”) AND “miller brewing company”	558	200	16
(“John D Bowlin” OR “Jack D Bowlin” OR “John Bowlin” OR “Jack Bowlin”) AND “miller brewing company”	470	200	34
“Patricia McKeithan” OR “Patti McKeithan”	239	239	29
(“Timothy Scully” OR “Tim Scully”) AND Miller	206	206	14
“William Schmus” OR “Bill Schmus”	173	173	17
“Marc S Firestone” OR “Marc Firestone”	477	200	5
“Tina Walls” AND “miller brewing company”	146	146	6
“Kathleen D Ryan” OR “Kathleen Ryan” OR “Kath Ryan” OR “Kath d Ryan”	189	189	13
“Yvonne Lumsden-Dill”	203	203	9

## Data Availability

Not applicable.

## Abbreviations

MBC: Miller Brewing Company
PM: Phillip Morris
ICAP: International Center for Alcohol Policies
WHO: World Health Organisation
CA: Corporate Affairs
WRA: Worldwide Regulatory Affairs
PMI: PM International
VP: Vice President
PAC: Political Action Committees
WRO: Washington Relations Office
CSR: Corporate social responsibility
